# Supplementary material for: A custom-made AAV1 variant (AAV1-T593K) enables efficient transduction of Japanese quail neurons in vitro and in vivo
Source: Commun Biol. 2023 Mar 28;6:337. doi: 10.1038/s42003-023-04712-6 (PMC10050006; doi:10.1038/s42003-023-04712-6)
Supplement: Supplementary file 2 — Supplementary Information [file 42003_2023_4712_MOESM2_ESM.pdf]

Supplementary Figure 1

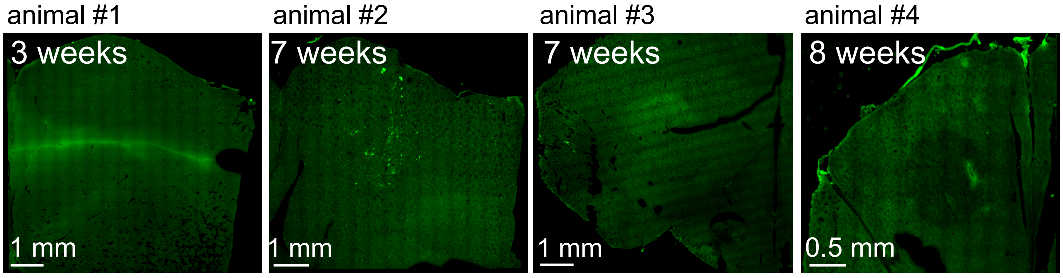

Supplementary Figure 1. AAV1 poorly infects quails' neurons in vivo. Injection of AAV2/1wt (AAV1 in brief) produced in our lab and expressing CAG-eYFP, into the Wulst of adult quails shows very poor infection efficiency. Frozen brain sections produced from four AAV1-injected adult quails taken at three, seven and eight weeks following injections. Only one animal showed infected cells.

Supplementary Figure 2

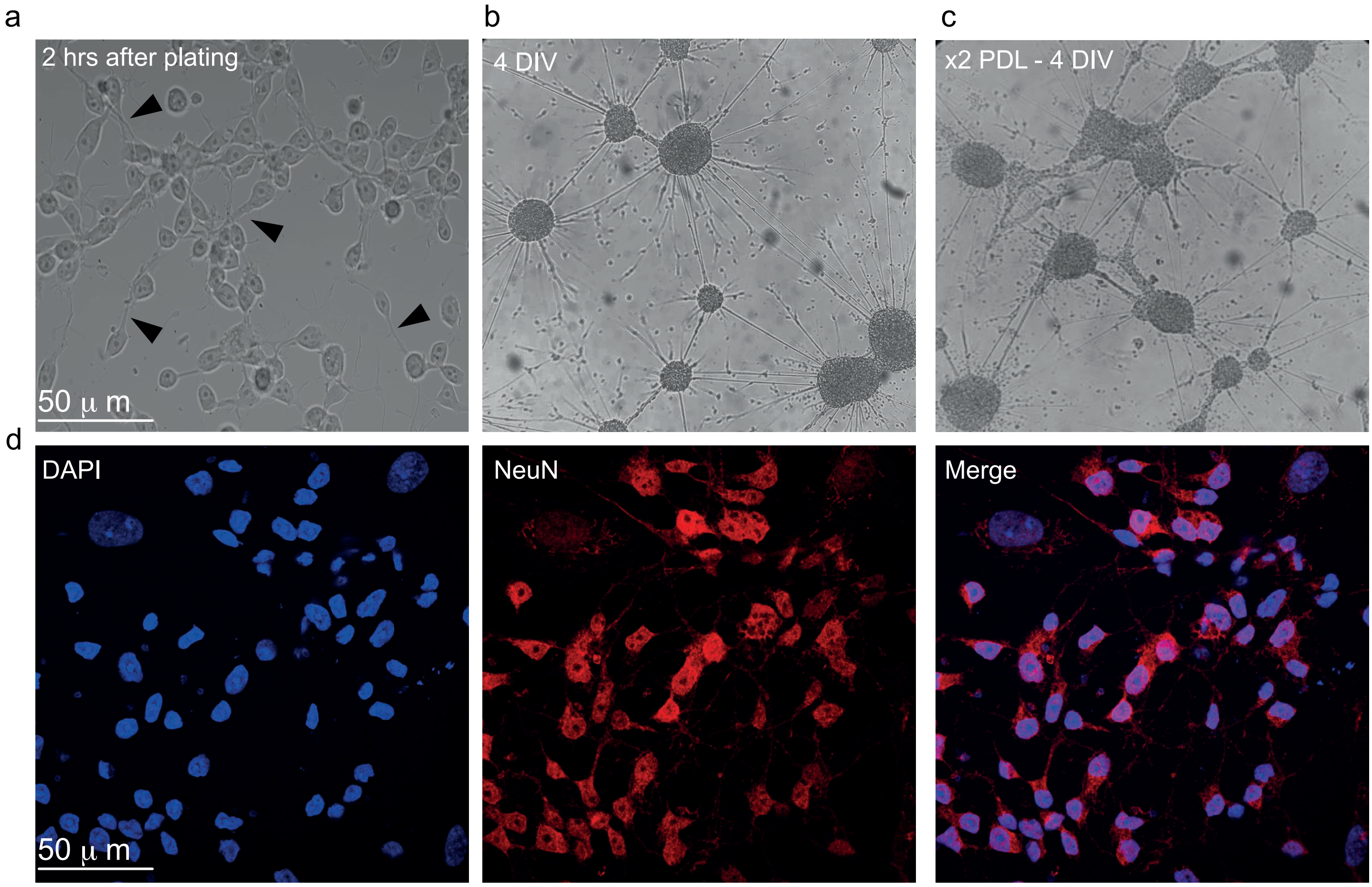

Supplementary Figure 2. Cultured quail cells do not adhere well to glass coversplis. a. Viable cells in culture (arrowheads) can be observed after two hours after disscotiation and plating, but culture do not survive past four DIV (b), or when using twofold higher concentrations of PDL (c). d. NeuN-staining (red) of cultured quail cells shows high abundance of NeuN-positive cells (DAPI, blue).

Supplementary Figure 3

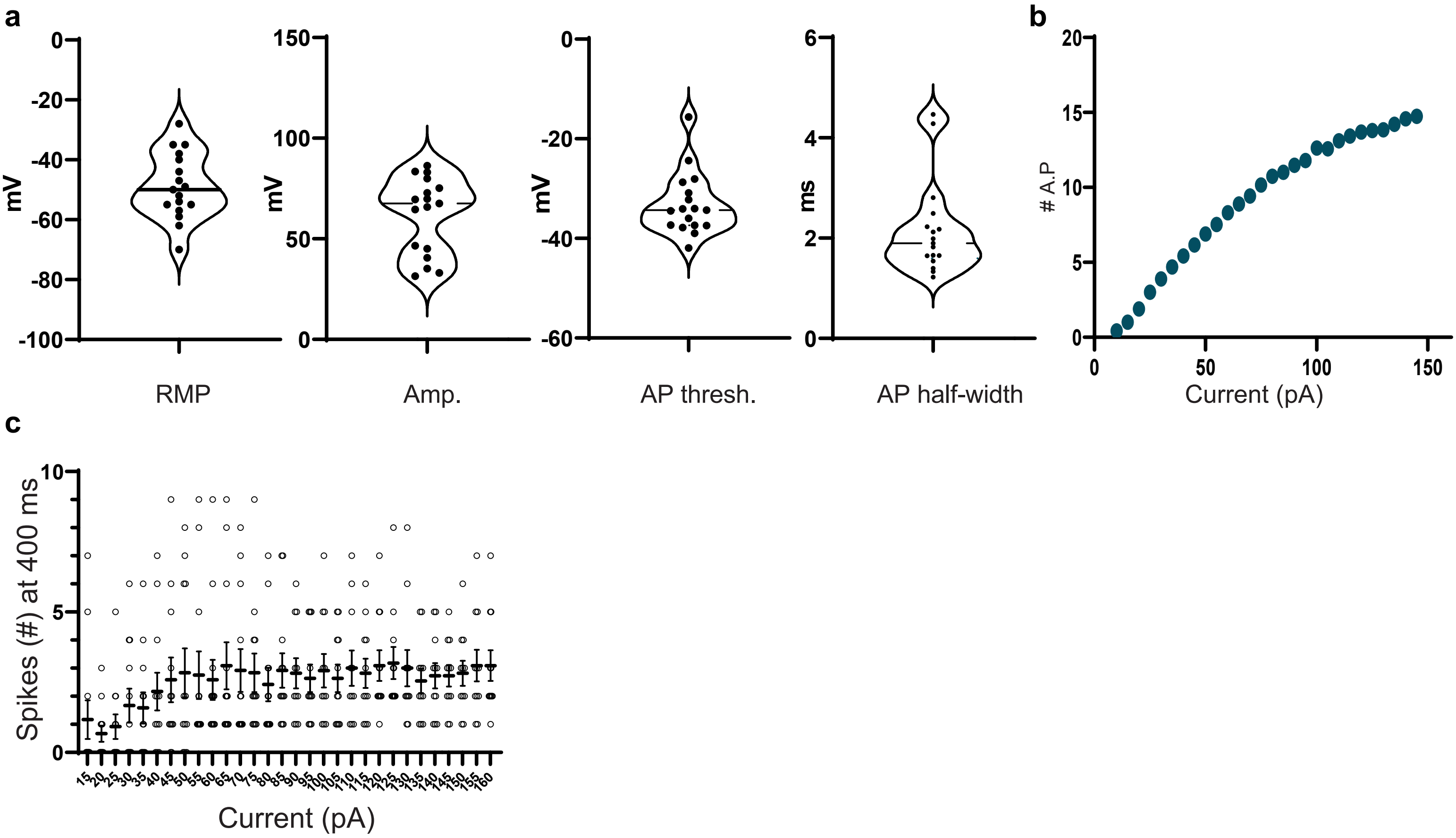

Supplementary Figure 3. Electrophysiological properties of embryonic mouse neurons. a. Resting membrane potential (RMP), action potential amplitude (Amp), action potential thereshold (AP thresh.) and half-width (AP half-width) are shown (n=19). b. Intrinsic excitability (as shown in Fig. 2). c. Intrinsic quail's neuronal excitability - individual data points for each current (as shown in Fig.2d).

Supplementary Figure 4

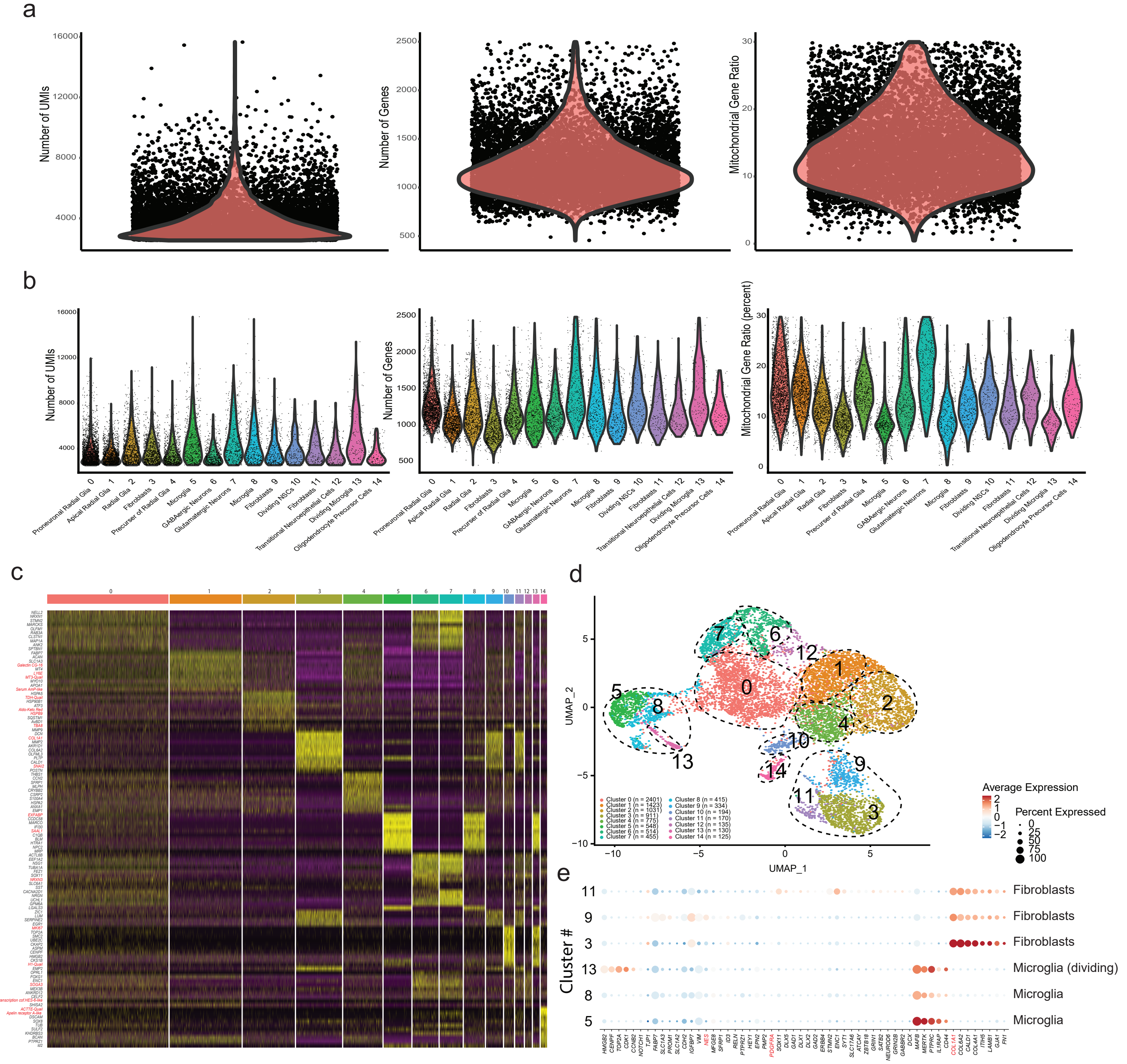

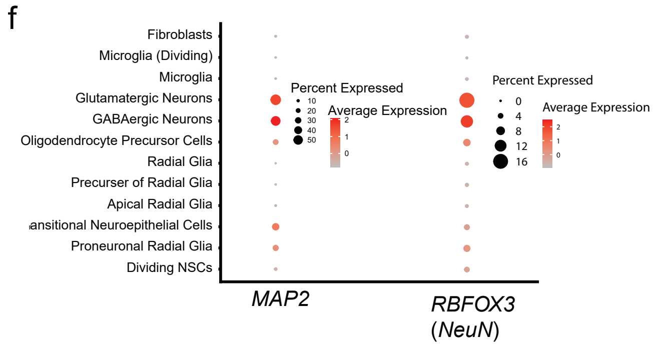

Supplementary Figure 4. Single cell mRNA sequencing of cultured cells reveals a large number of cellular populations. a, b. Violin plots of the number of UMIs, number of genes and mitochondrial gene ratio (10-20%) for all cells (a) and per cluster (b) are shown. c. Classification of clusters based on top 200 differentially expressed genes (DEGs). d, e. subclusters of fibroblasts, and microglia reveal various subtypes. f. Dotplots showing differential expression of MAP2 and RBFOX3 (NeuN) among the different clusters.

Supplementary Figure 5

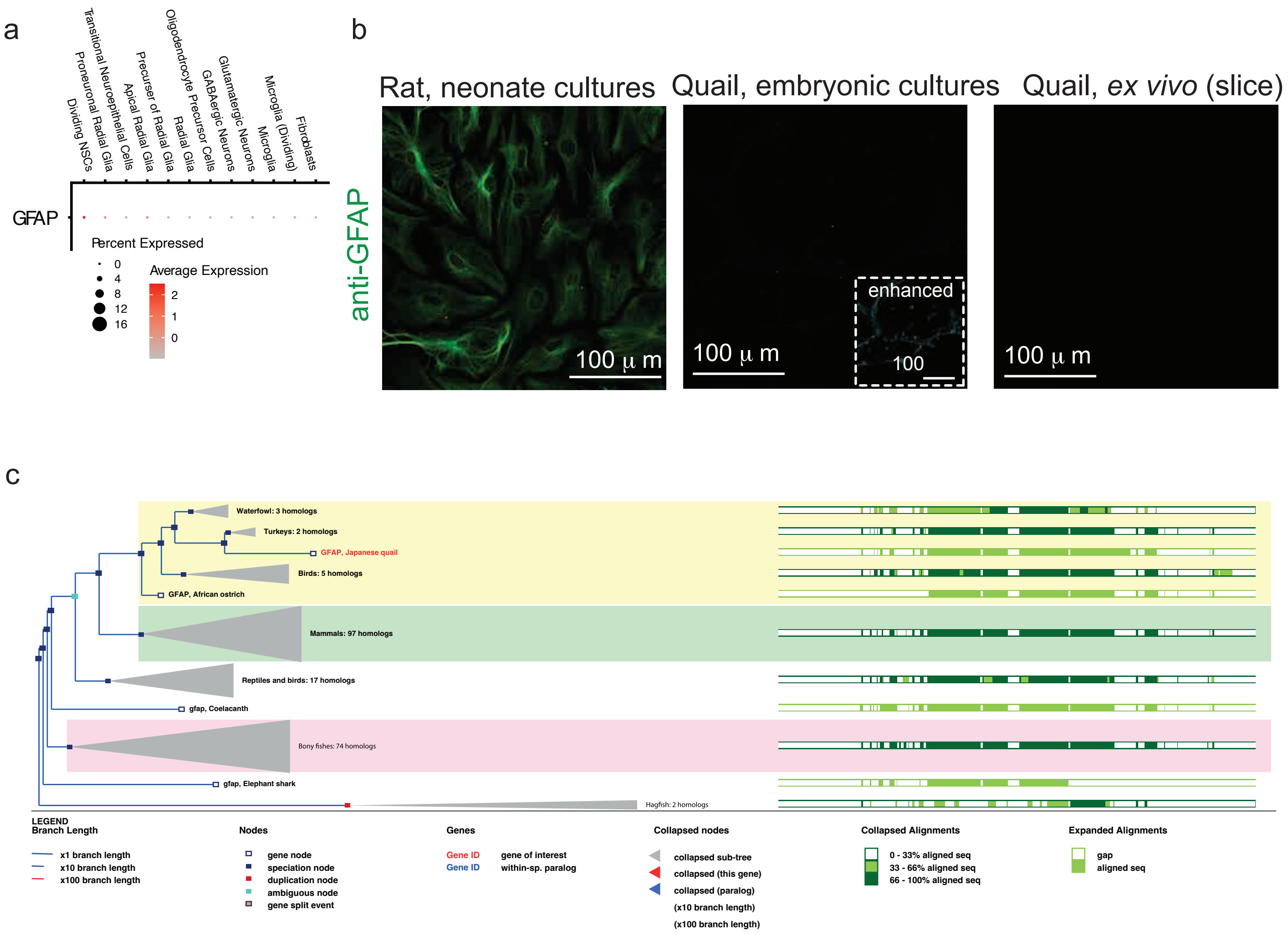

Supplementary Figure 5. Quails cultures show no GFAP expression in single cell mRNA sequencing and Immunohistochemistry. a. Dotplot showing (lack of) GFAP expression in our sc. mRNA sequencing among the clusters. b. Immunostaining for GFAP (green) in cultures taken from rat neonates (left) and quail embryonic cultures (middle), and quail brain slices ex vivo (right). Note the complete lack of staining against GFAP in quails. c. Phylogenetic tree of GFAP across species. Note the large distance of this gene between mammals and birds.

Supplementarty Figure 6

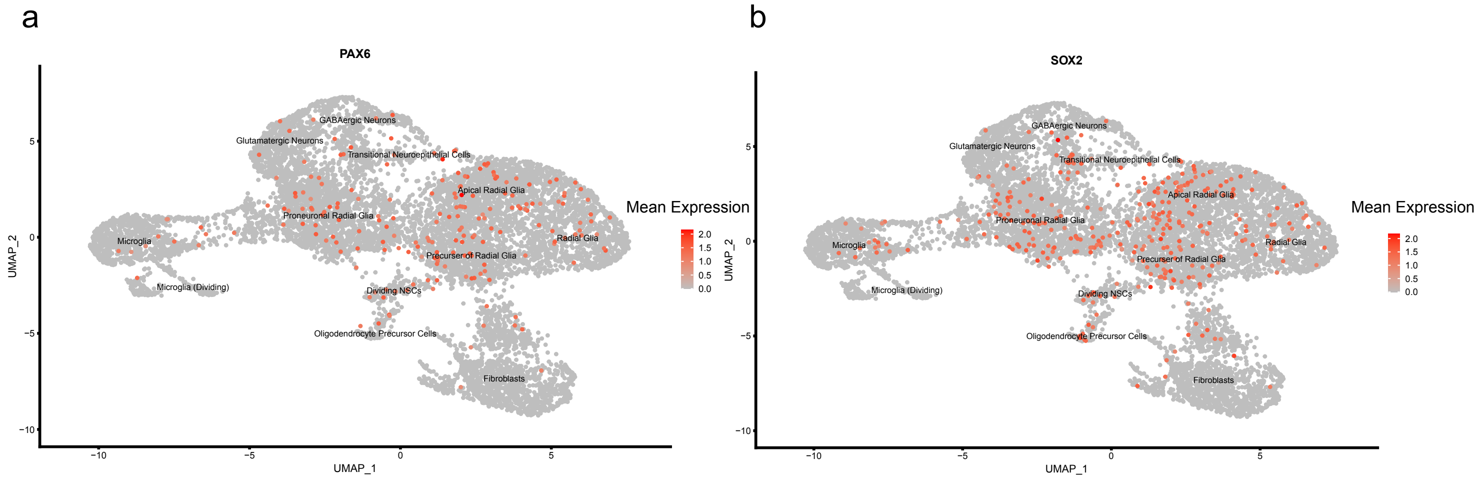

Supplementary Figure 6. Progenitor markers in cultured cells. Feature plots showing the expression of PAX6 (a left) and SOX2 (right b)— early developmental marker genes in quail cultures.

Supplementary Figure 7

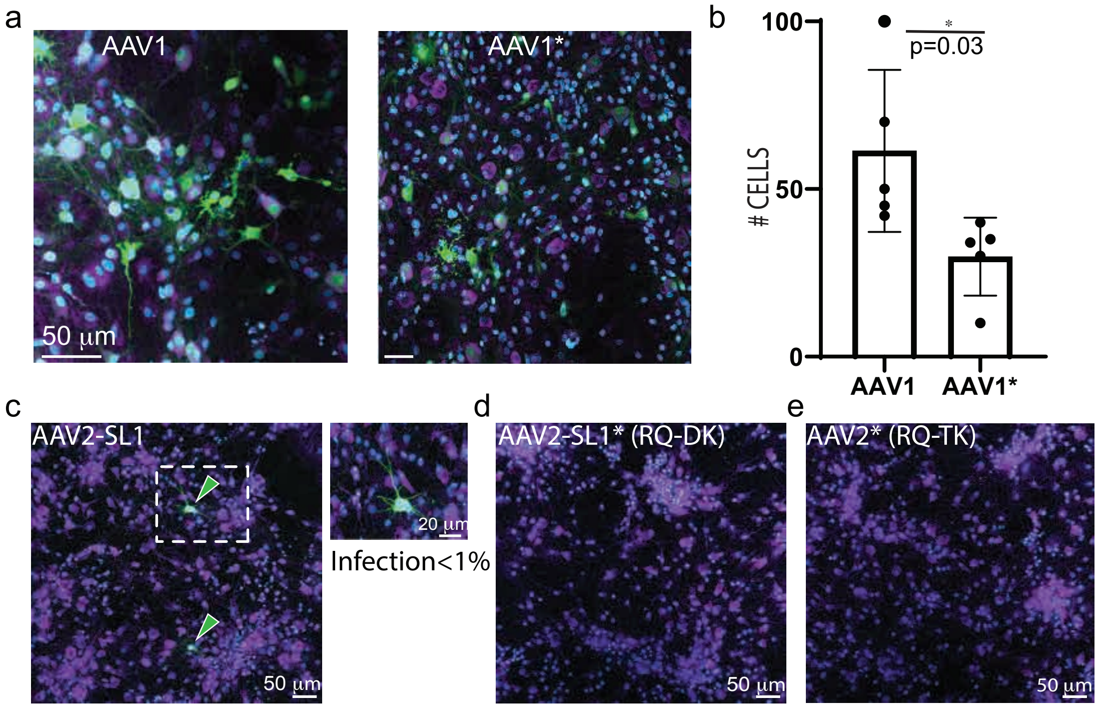

Supplementary Figure 7. Transduction of rat primary neuronal cultures by AAV1*, and quails cultures by AAV2 WT and variants. a. AAV1* shows twofold reduction in infection efficiency of rat primary neurons in comparison to AAV1 (AAV1*- (mean ± SEM) - 29.8±5.2 compared to AAV1 61.4 ± 10.8 cells/frame, T-test, p=0.03), summarized in (b). c. AAV2-SL1 poorly infects quail cultures, with less than 1% infection of cells (green arrowheads and inset). d. AAV2 variants (left micrograph-AAV2-SL1*, right micrgraph AAV2*) are completely non-infectious (magenta-NeuroTrace staining; blue- DAPI staining).

Supplementary Figure 8

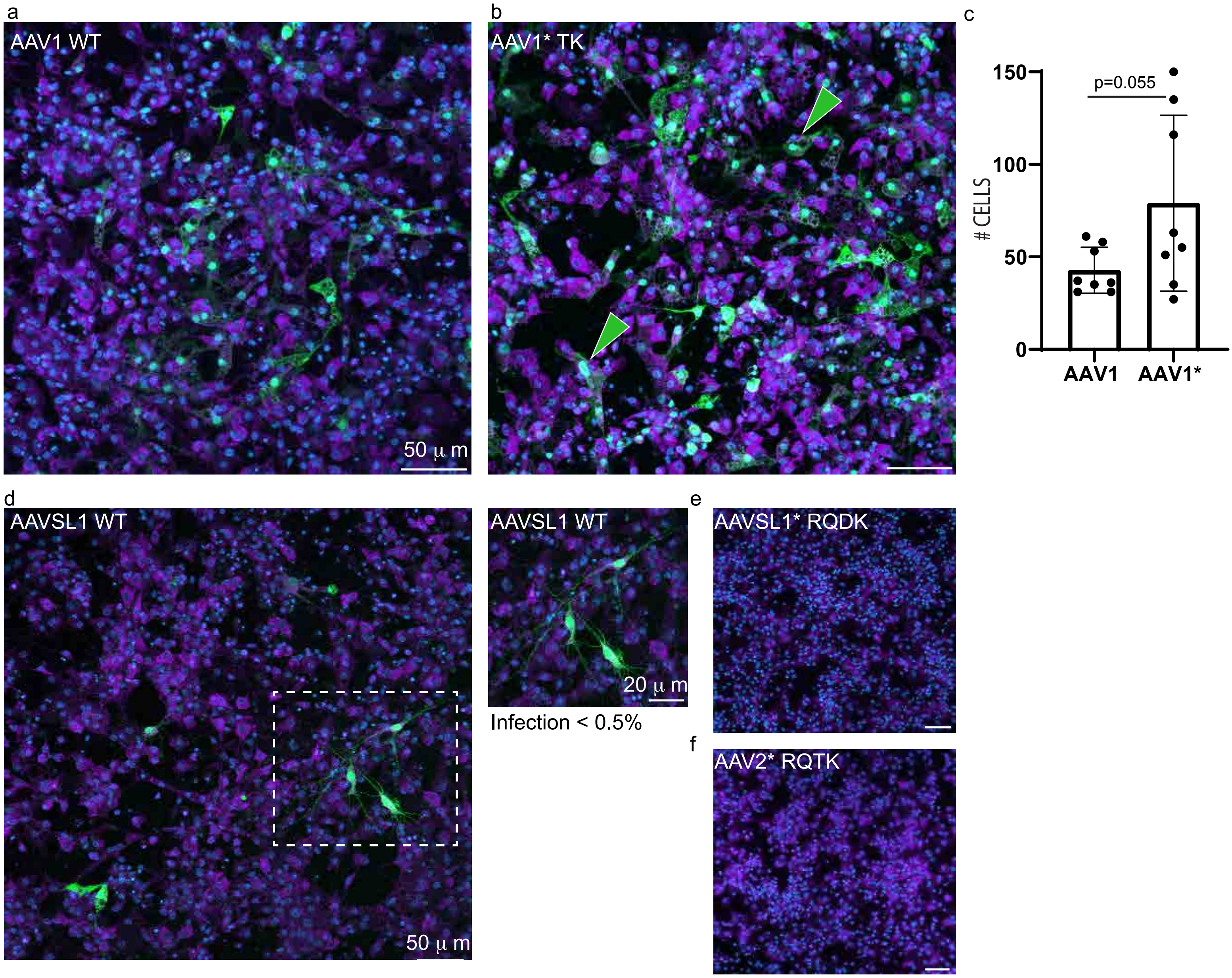

Supplementary Figure 8. AAV1* variant show improved tranduction of chicken (gallus gallus) cultures, in vitro. a, b. Micrographs of primary chicken cultures produced from embryonic brains, infected with AAV1 (a) and AAV1* (b) expressing eYFP (42.7 ± 4.4, 79 ±16.7, respectively, T-test, p=0.055); summarized in c. In b, green arrowheads show transduced neurons (NeuroTrace-positive, magenta; DAPI-blue). d. AAV2-SL1 poo rly infects cultured chicken brain cells. Inset shows YFP-expressing neurons (green). e, f. AAV2 varinats (AAV2-SL1* and AAV2*) lose ability to infect cultures.

Supplementary Figure 9

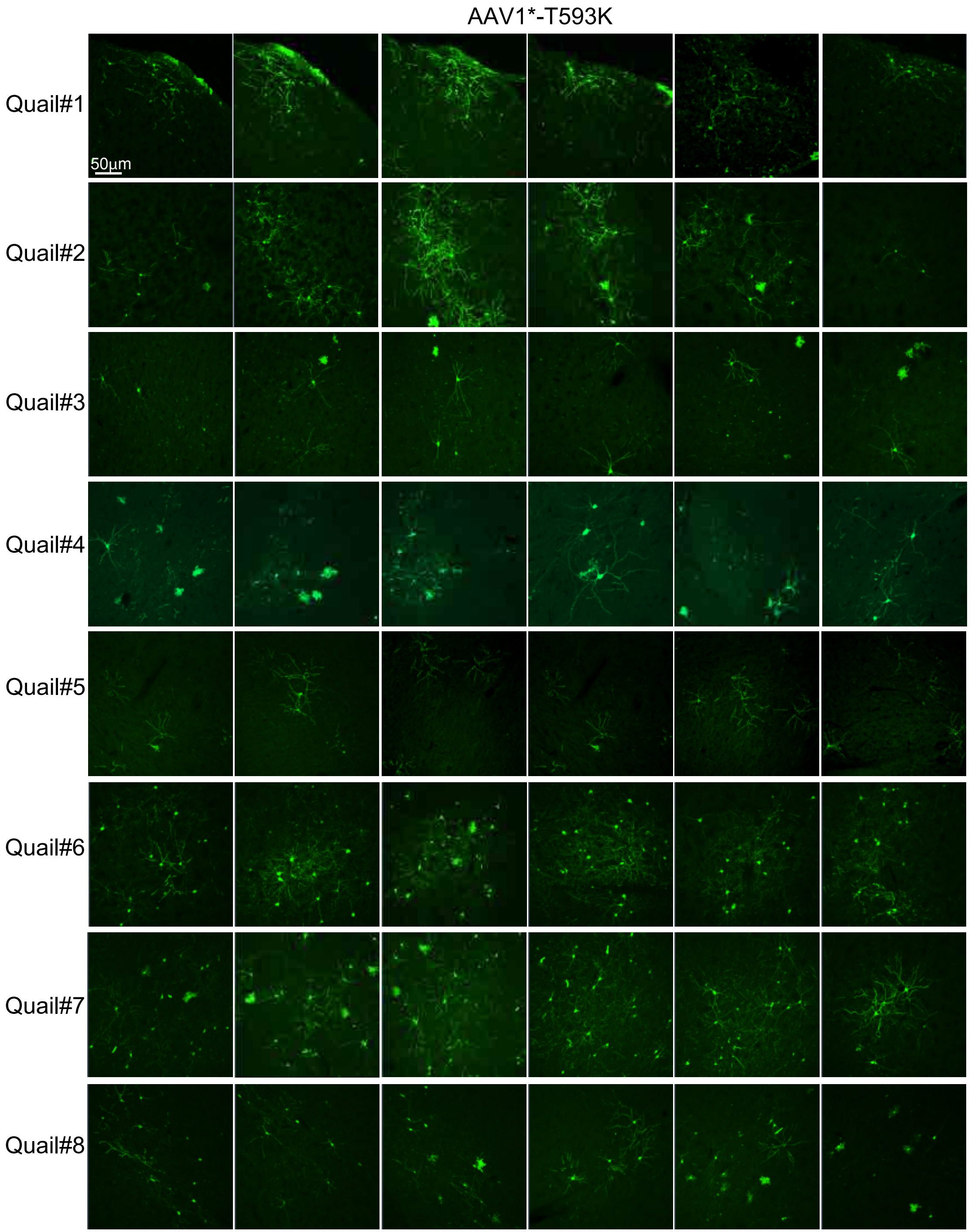

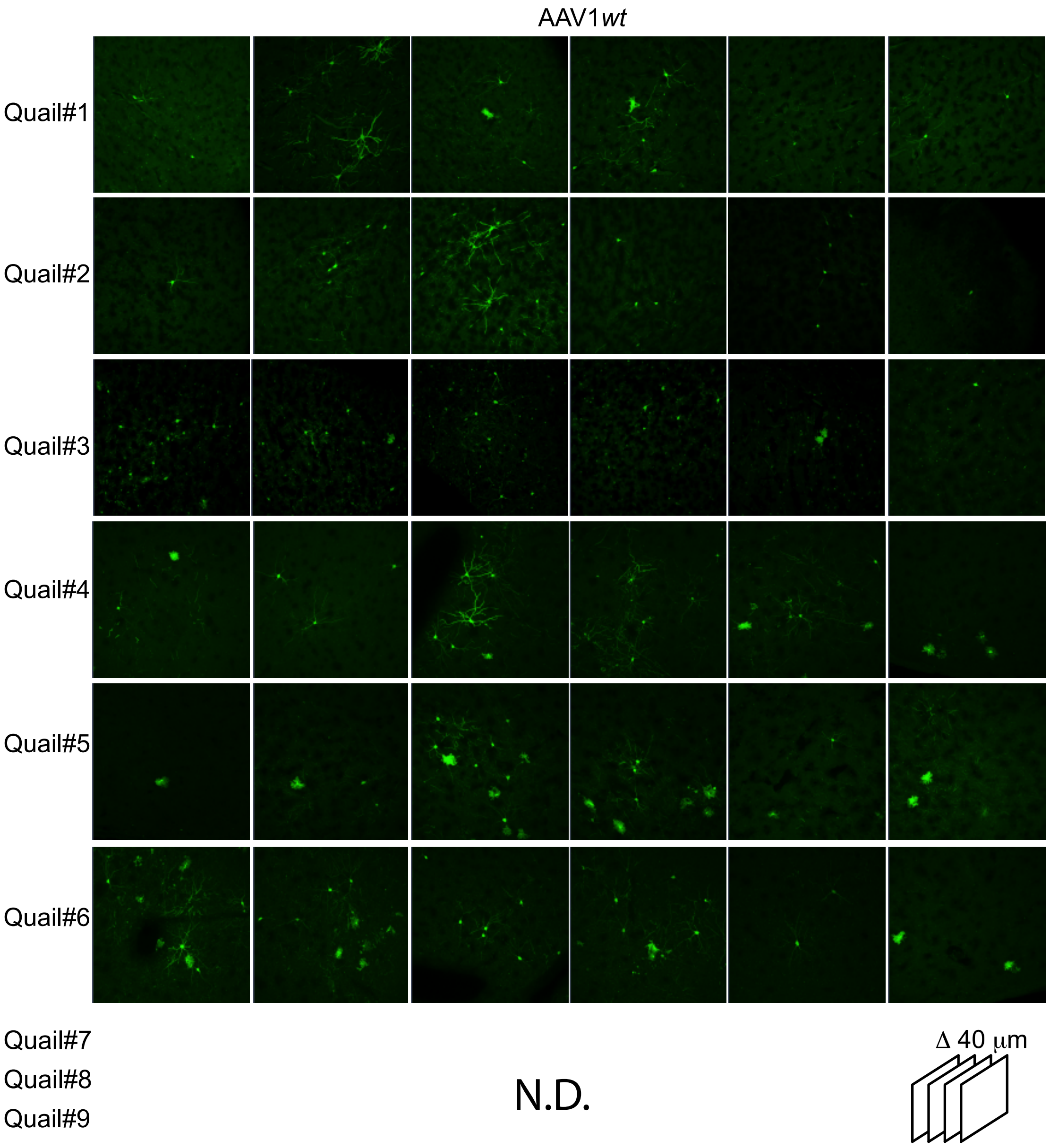

Supplementary Figure 9. AAV1* shows improved tranduction of quail brains *in vivo*. Complete dataset of sequential coronal brain slices (every 40 μm) produced after quails have been injected by AAV1* (top) or AAV1 (bottom) expressing CAG-eYFP or CAG-GCaMP6. Three quails in which we found no expression are noted at bottom (#7-9) (slices shown in Suppl. 1).

Supplementary Figure 10

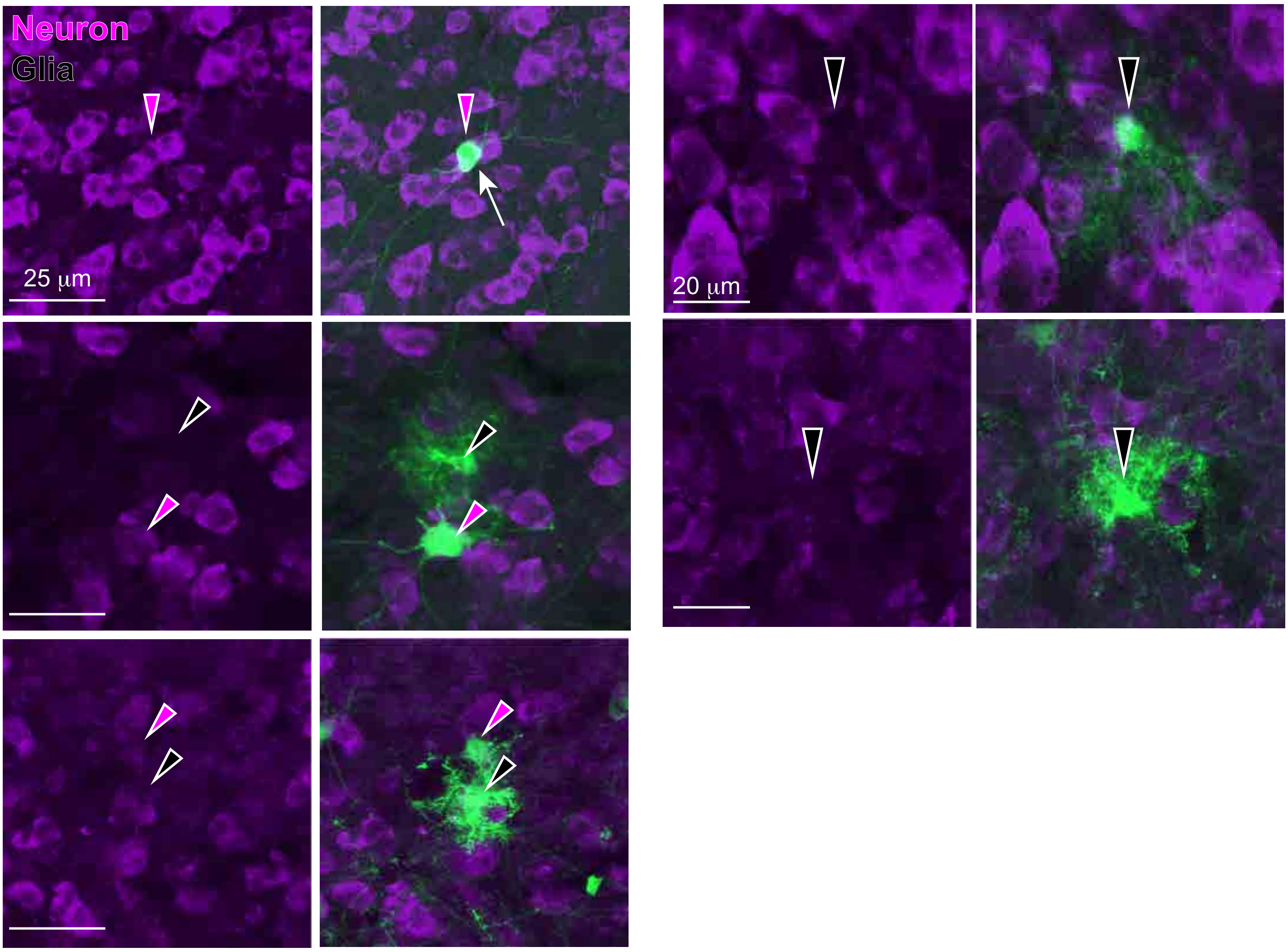

Supplementary Figure 10. NeuroTrace staining of quail brain slices *in vivo*.
High power micrographs showing that YFP-positive and NeuroTrace-positive cells (pink arrowheads) have prototypical neuronal morphology, whereas YFP-positive and NeuroTrace-negative (black arrowheads) exhibit multiple concentric and intact processes, highly reminiscent of astrocytes.

Supplementary Figure 11

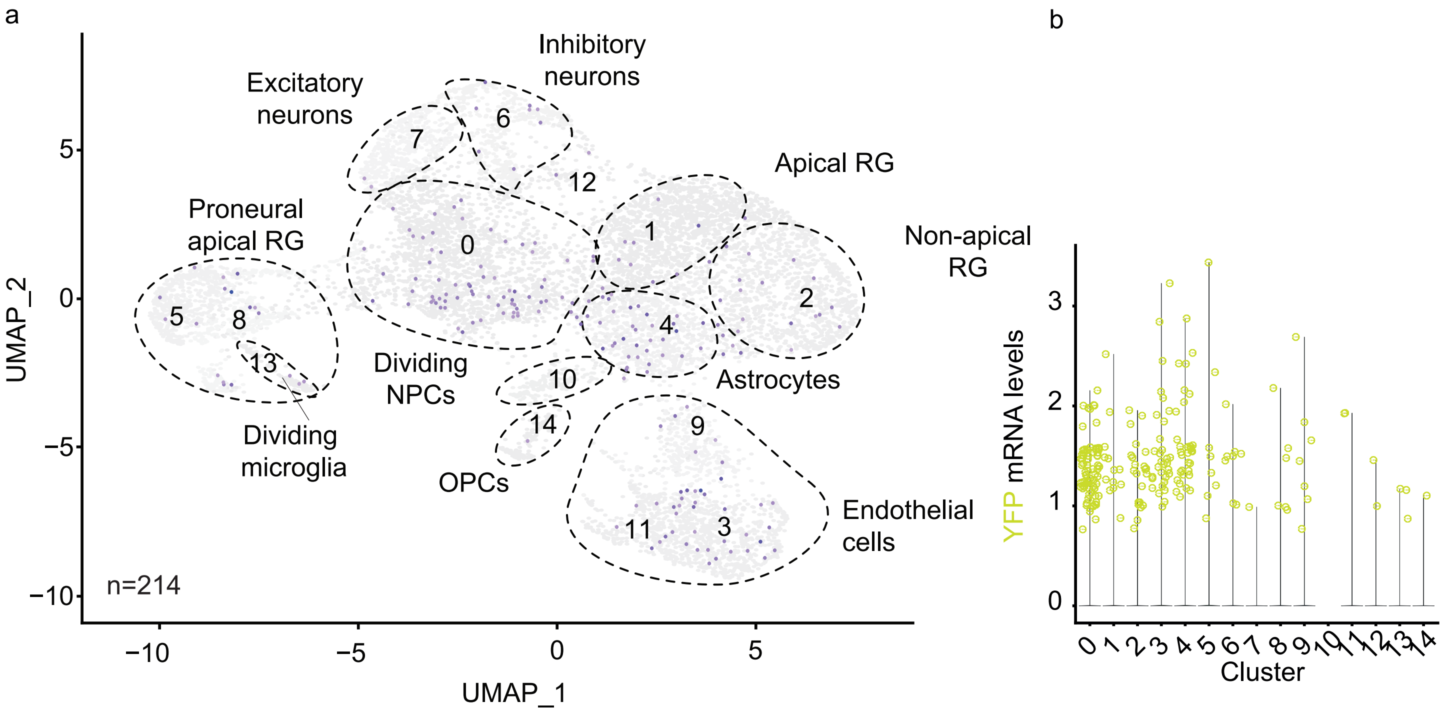

Supplementary Figure 11. AAV1* infects various cells types with no correlation ot presence of AAVR. Feature map of cells expressing eYFP across various clusters (a). mRNA levels are depicted (b).

Supplementary Table 1. Comparison between different culturing protocols for brain cells.

	Mammals (Rats/mouse)[1]	Chicken[2]	Quails (here)
**Proteolytic enzyme and conditions**	0.5% trypsin, 15 min. at 37 °C (water bath)	0.25% trypsin, 10-15 min. at 37 °C (CO_2 incubator)	30 U/ml Papain, 30 min. at 37 °C (water bath)
**Dissection medium**	HBSS + HEPES	DMEM + 10% FBS	DMEM
**Neuronal growth medium**	MEM (Minimal Essential Media)<br>5% FBS<br>2% B27<br>1% Glutamax<br>2% D-glucose<br>0.1% serum extender	NBM (Neurobasal Media)<br>2% B27<br>2 mM Glutamax<br>20 ng/ml NGF<br>X% Pen/Strep	NBM (Neurobasal Media)<br>2% B27<br>0.25% Glutamax<br>1% Pen/Strep
**Growth surface & coating**	Glass coverslips<br>0.5 mg/ml PLL	Glass coverslips<br>0.1 mg/ml PDL	60 mm tissue culture treated plates<br>2 mg/ml PDL

Supplementary Table 2. Cell markers for classification of clusters.

Cell type	Markers
**Fibroblasts**	COL1A1, COL4A1, CALD1, ITIH5, COL6A2, LAMB1[3,4]
**Microglia**	PTPRC, MAFB, IL1RAP, CD44, MERTK[5]
**Neuronal progenitor stem cells**	NOTCH1, TOP2A, HMGB2, CENPF, CDK1, CCNB2, TJP1, CDH2, IGFBP7, VIM, NES, SFRP1, RELN, MFGE8, FABP7, ID3[5–8]
**Oligodendrocyte progenitor cells**	PTPRZ1, HEY1, EPN2, PMP2, PDGFRA, OLIG2[5]
**Precursor of radial glia**	SLC1A3, PROM1, SLC1A2, FN1, CDH2, IGFBP7, VIM, NES, SFRP1, RELN, MFGE8, GJA1, FABP7, ID3[9,10]
**Glutamatergic neurons**	STMN2, SLC17A6, ENC1, SYT1, ATCAY, ZBTB18, GRIN1, SATB2, NEUROD6, GRIN2B, DCX[11]
**GABAergic neurons**	GAD1, GAD2, DLX2,1,5, SOX11, ERBB4, STMN2, ENC1, SYT1[12]
**Pro-neuronal Radial glia**	SLC1A3, FABP7, VIM, NES, PROM1, SLC1A2, CDH2, IGFBP7, MFGE8, STMN2, NEUROD1[10]
**Radial glia**	CDH2, IGFBP7, VIM, NES [10]
**Apical RG**	SLC1A3, FABP7, VIM, NES, PROM1, SLC1A2, CDH2, IGFBP7[10]
**Intermediate proneuronal radial glia**	CDH2, IGFBP7, VIM, NES, SOX11, ENC1[13]

Supplementary Table 3. Interacting pairs of residues between hAAVR and qAAVR with capsids of AAV1 and AAV2.

Human AAVR residues	Quail AAVR residues	AAV1 capsid residues[14]	AAV2 capsid residues[15]
**S413**	S	-	E499
**P414**	P	-	E499
**S425**	S	D590	Q589
**T426**	T	D590	Q589
**V427**	V	T504	Q589, T503
**D429**	D	W503, T504	W502, T503
**S431**	S	W503	S267, W502
**Q432**	R172	S268, W503	R471
**S433**	S	S268, N269	S267
**T434**	T	G266, A267, S268	G265, A266, S267
**D435**	D	G266, A267, H272	G265, A266, H271
**D436**	D	A263, H272, S385	Q263, H271, S384
**D437**	D	H272, S385, Q386	H271, S384, Q385
**K438**	K	N269, N383, G384	N268, H271, N382, D528
**I439**	I	N269	N268
**Y442**	Y	N269	-
**I462**	I	-	K507
**K464**	T204	T593	T592

*** residues in red are those targeted by point mutations in AAV1/AAV2 capsid plasmid.**

Supplementary Table 4. YFP expression spread *in vivo*.

Variant	Distribution in 'z'
**AAV1**	270 - 450 μm
**AAV1*** (T593K)	360 - 540 μm

Supplementary Table 5. In vivo viral injections information

Quail number	AAV1 serotype	Gene	Age	Time	Expression	volume
#1	WT	YFP	2 months (Adult)	3 weeks	N.D	0.5 ul
#2	WT	YFP	2 months (Adult)	7 weeks	N.D	0.5 µl
#3	WT	YFP	2 months (Adult)	7 weeks	200.2 cells/mm2	0.5 µl
#4	WT	YFP	2 months (Adult)	8 weeks	N.D	0.5 µl
#5	WT	YFP	4 weeks (young)	8 weeks	80.7 cells/mm2	1 µl
#6	WT	YFP	4 weeks (young)	7 weeks	81.2 cells/mm2	1 µl
#7	WT	YFP	4 weeks (young)	6 weeks	174.9 cells/mm2	1 µl
#8	WT	YFP	4 weeks (young)	6 weeks	94.88 cells/mm2	1 µl
#9	WT	YFP	4 weeks (young)	6 weeks	77.777 cells/mm2	1 µl
#1	T593K	YFP	2 months (Adult)	7 weeks	376.8 cells/mm2	0.5 µl
#2	T593K	YFP	2 months (Adult)	8 weeks	922.6 cells/mm2	0.5 µl
#3	T593K	Gcamp	2 months (Adult)	8 weeks	420.3 cells/mm2	0.5 µl
#4	T593K	YFP	4 weeks (young)	7 weeks	658.8 cells/mm2	1 µl
#5	T593K	YFP	4 weeks (young)	7 weeks	203.76 cells/mm2	1 µl
#6	T593K	YFP	4 weeks (young)	6 weeks	805.555 cells/mm2	1 µl
#7	T593K	YFP	4 weeks (young)	6 weeks	527.777 cells/mm2	1 µl
#8	T593K	YFP	4 weeks (young)	6 weeks	349.999 cells/mm2	1 µl

Supplementary Table 6. Point mutation primers for PCR information.

Mutation	Sense primer	Antisense primer
**pAAV2/2 and SL1 R471Q**	5′CAGGCCGGAGCGAGTGACATTCA GGACCAGTCTAGGAACTGGCTT	5′AAGCCAGTTCCTAGACTGGTCCTG AATGTCACTCGCTCCGGCCTG
**pAAV2/2 T592K**	5′AGAGGCAACAGACAAGCAGCTAA AGCAGATGTCAACACACAAGGC	5′GCCTTGTGTGTTGACATCTGCTTTA GCTGCTTGTCTGTTGCCTCT
**SL1 D592K**	5′AGAGGCAACCTAGCAGACCAAAAA TACACAAAAACTGCTAGGCAA	5′TTGCCTAGCAGTTTTTGTGTATTTTT GGTCTGCTAGGTTGCCTCT
**pAAV2/1-T593K**	5′AGCAGCAGCACAGACCCTGCGAA AGGAGATGTGCATGCTATGGGA	5′TCCCATAGCATGCACATCTCCTTTC GCAGGGTCTGTGCTGCTGCT

Supplementary References

1. Berlin, S. & Isacoff, E. Optical Control of Glutamate Receptors of the NMDA-Kind in Mammalian Neurons, with the Use of Photoswitchable Ligands. in *Neuromethods* 293–325 (2018). doi:10.1007/978-1-4939-7228-9_10.
2. Kumar, M., Keller, B., Makalou, N. & Sutton, R. E. Systematic Determination of the Packaging Limit of Lentiviral Vectors. *Hum. Gene Ther.* **12**, 1893–1905 (2001).
3. Su, C. *et al.* Single-Cell RNA Sequencing in Multiple Pathologic Types of Renal Cell Carcinoma Revealed Novel Potential Tumor-Specific Markers. *Front. Oncol.* **11**, 719564 (2021).
4. Skelly, D. A. *et al.* Single-Cell Transcriptional Profiling Reveals Cellular Diversity and Intercommunication in the Mouse Heart. *Cell Rep.* **22**, 600–610 (2018).
5. Zhong, S. *et al.* A single-cell RNA-seq survey of the developmental landscape of the human prefrontal cortex. *Nature* **555**, 524–528 (2018).
6. Tirosh, I. *et al.* Dissecting the multicellular ecosystem of metastatic melanoma by single-cell RNA-seq. *Science* **352**, 189–196 (2016).
7. Eze, U. C., Bhaduri, A., Haeussler, M., Nowakowski, T. J. & Kriegstein, A. R. Single-cell atlas of early human brain development highlights heterogeneity of human neuroepithelial cells and early radial glia. *Nat. Neurosci.* **24**, 584–594 (2021).
8. Kaczmarczyk, L. *et al.* Slc1a3-2A-CreERT2 mice reveal unique features of Bergmann glia and augment a growing collection of Cre drivers and effectors in the 129S4 genetic background. *Sci. Rep.* **11**, 5412 (2021).
9. Zeisel, A. *et al.* Cell types in the mouse cortex and hippocampus revealed by single-cell RNA-seq. *Science* **347**, 1138–1142 (2015).
10. Johnson, M. B. *et al.* Single-cell analysis reveals transcriptional heterogeneity of neural progenitors in human cortex. *Nat. Neurosci.* **18**, 637–646 (2015).
11. Chen, R., Wu, X., Jiang, L. & Zhang, Y. Single-Cell RNA-Seq Reveals Hypothalamic Cell Diversity. *Cell Rep.* **18**, 3227–3241 (2017).
12. Fan, X. *et al.* Spatial transcriptomic survey of human embryonic cerebral cortex by single-cell RNA-seq analysis. *Cell Res.* **28**, 730–745 (2018).

13. Haslinger, A., Schwarz, T. J., Covic, M. & Lie, D. C. Expression of Sox11 in adult neurogenic niches suggests a stage-specific role in adult neurogenesis. *Eur. J. Neurosci.* **29**, 2103–2114 (2009).
14. Zhang, R. *et al.* Divergent engagements between adeno-associated viruses with their cellular receptor AAVR. *Nat. Commun.* **10**, 3760 (2019).
15. Zhang, R. *et al.* Adeno-associated virus 2 bound to its cellular receptor AAVR. *Nat. Microbiol.* **4**, 675–682 (2019).
