# Supplementary material for: A custom-made AAV1 variant (AAV1-T593K) enables efficient transduction of Japanese quail neurons in vitro and in vivo
Source: Commun Biol. 2023 Mar 28;6:337. doi: 10.1038/s42003-023-04712-6 (PMC10050006; doi:10.1038/s42003-023-04712-6)
Supplement: Supplementary file 3 — Reporting Summary [file 42003_2023_4712_MOESM3_ESM.pdf]

nature portfolio

Corresponding author(s): Shai Berlin

Last updated by author(s): Jan 23, 2023

Reporting Summary

Nature Portfolio wishes to improve the reproducibility of the work that we publish. This form provides structure for consistency and transparency in reporting. For further information on Nature Portfolio policies, see our Editorial Policies and the Editorial Policy Checklist.

Statistics

For all statistical analyses, confirm that the following items are present in the figure legend, table legend, main text, or Methods section.

n/a	Confirmed	
☐	☒	The exact sample size (*n*) for each experimental group/condition, given as a discrete number and unit of measurement
☐	☒	A statement on whether measurements were taken from distinct samples or whether the same sample was measured repeatedly
☐	☒	The statistical test(s) used AND whether they are one- or two-sided<br>*Only common tests should be described solely by name; describe more complex techniques in the Methods section.*
☐	☒	A description of all covariates tested
☐	☒	A description of any assumptions or corrections, such as tests of normality and adjustment for multiple comparisons
☐	☒	A full description of the statistical parameters including central tendency (e.g. means) or other basic estimates (e.g. regression coefficient) AND variation (e.g. standard deviation) or associated estimates of uncertainty (e.g. confidence intervals)
☐	☒	For null hypothesis testing, the test statistic (e.g. *F*, *t*, *r*) with confidence intervals, effect sizes, degrees of freedom and *P* value noted<br>*Give P values as exact values whenever suitable.*
☒	☐	For Bayesian analysis, information on the choice of priors and Markov chain Monte Carlo settings
☒	☐	For hierarchical and complex designs, identification of the appropriate level for tests and full reporting of outcomes
☒	☐	Estimates of effect sizes (e.g. Cohen's *d*, Pearson's *r*), indicating how they were calculated

Our web collection on statistics for biologists contains articles on many of the points above.

Software and code

Policy information about availability of computer code

Data collection	Data was collected using a commercial confocal microscope (zeiss, Germany).The RNAseq data was generated on Illumina NextSeq2000, P2 100 cycles<br>(R1-28bp, R2-90bp, I1-10bp, I2-10bp) (Illumina, cat no. 20046811).
Data analysis	Confocal images were analysed using Zen software, single cell mRNA seq. data were analyzed using R (Version 4.0.1) and Seurat R package. Statistics: sigmaplot, prism

For manuscripts utilizing custom algorithms or software that are central to the research but not yet described in published literature, software must be made available to editors and reviewers. We strongly encourage code deposition in a community repository (e.g. GitHub). See the Nature Portfolio guidelines for submitting code & software for further information.

Data

Policy information about availability of data

All manuscripts must include a data availability statement. This statement should provide the following information, where applicable:

- Accession codes, unique identifiers, or web links for publicly available datasets
- A description of any restrictions on data availability
- For clinical datasets or third party data, please ensure that the statement adheres to our policy

All data generated or analysed during this study are included in the article and its Supplementary Information files. Raw single cell mRNA seq data is placed in a public data depository (NCBI GEO: GSE227334) and a data availability statement is included in the text.

Human research participants

Policy information about studies involving human research participants and Sex and Gender in Research.

Reporting on sex and gender	*Use the terms sex (biological attribute) and gender (shaped by social and cultural circumstances) carefully in order to avoid confusing both terms. Indicate if findings apply to only one sex or gender; describe whether sex and gender were considered in study design whether sex and/or gender was determined based on self-reporting or assigned and methods used. Provide in the source data disaggregated sex and gender data where this information has been collected, and consent has been obtained for sharing of individual-level data; provide overall numbers in this Reporting Summary. Please state if this information has not been collected. Report sex- and gender-based analyses where performed, justify reasons for lack of sex- and gender-based analysis.*
Population characteristics	*Describe the covariate-relevant population characteristics of the human research participants (e.g. age, genotypic information, past and current diagnosis and treatment categories). If you filled out the behavioural & social sciences study design questions and have nothing to add here, write "See above."*
Recruitment	*Describe how participants were recruited. Outline any potential self-selection bias or other biases that may be present and how these are likely to impact results.*
Ethics oversight	*Identify the organization(s) that approved the study protocol.*

Note that full information on the approval of the study protocol must also be provided in the manuscript.

Field-specific reporting

Please select the one below that is the best fit for your research. If you are not sure, read the appropriate sections before making your selection.

☒ Life sciences ☐ Behavioural & social sciences ☐ Ecological, evolutionary & environmental sciences

For a reference copy of the document with all sections, see nature.com/documents/nr-reporting-summary-flat.pdf

Life sciences study design

All studies must disclose on these points even when the disclosure is negative.

Sample size	Each datum point in the plots represents one cell or one animal (depending on results). Unpaired two tailed t-test was used to compare between two groups and One-way ANOVA following Dunnet test was used to compare mulitple groups. IN vivo experiments included 5-8 animal in each group. Sample sizes used were chosen based on established parameters in the field.
Data exclusions	no data were excluded from analyses
Replication	Every experiment was repeated at least 3 times.
Randomization	Cultures were produced from embryos (of both sexes) from 7 to 9 DIO and gorwn for up to 21 days. Data were pooled from independent cultures of identical age in vitro.
Blinding	Animals sacrificed in this study were chosen randomlly.<br>Blinding was not relevant to cultures. All cultures and animals were imaged and examined under identical consitions.

Reporting for specific materials, systems and methods

We require information from authors about some types of materials, experimental systems and methods used in many studies. Here, indicate whether each material, system or method listed is relevant to your study. If you are not sure if a list item applies to your research, read the appropriate section before selecting a response.

Materials & experimental systems

n/a	Involved in the study
☐	☒ Antibodies
☐	☒ Eukaryotic cell lines
☒	☐ Palaeontology and archaeology
☐	☒ Animals and other organisms
☒	☐ Clinical data
☒	☐ Dual use research of concern

Methods

n/a	Involved in the study
☒	☐ ChIP-seq
☒	☐ Flow cytometry
☒	☐ MRI-based neuroimaging

Antibodies

Antibodies used

anti-NeuN antibody (mouse, clone A60, Millipore CAT# MAB377) with secondary antibody (Rhodamine Red-X goat Anti-muse IgG, Jackson
Laboratories, CAT# 115-295-003). GFAP-staining was performed by using anti-GFAP (mouse, clone G-A-5, Calbiochem, CAT# IF03L), followed by
staining with a secondary antibody (Alexa Fluor-488 goat anti-mouse IgG, Jackson Laboratories, CAT# 115-545-003).

Validation

Antibodies were validated by staining primary neurons from rats and only then for staining of the test cultures

Eukaryotic cell lines

Policy information about cell lines and Sex and Gender in Research

Cell line source(s)

Human Embryonic Kidney cells, ATCC #CRL-1573

Authentication

Cell lines were not authenticated as they were purchased from a validated source. Cell type does not affect the results of the paper, as most lines produced were primary cells. HOwever, partial validation of the identity of the HEK cells is provided
by the high titers of the viruses produced.

Mycoplasma contamination

All cell were tested negative to mycoplasma

Commonly misidentified lines
(See ICLAC register)

Name any commonly misidentified cell lines used in the study and provide a rationale for their use.

Animals and other research organisms

Policy information about studies involving animals; ARRIVE guidelines recommended for reporting animal research, and Sex and Gender in Research

Laboratory animals

We used Japanese quail embryos (7-9 days in ovo) , and juvenile/adult Japanese quails

Wild animals

the study did not involve wild animals

Reporting on sex

animals were randomly picked- females and males.

Field-collected samples

the study did not involve samples collected from the field

Ethics oversight

All animal procedure protocols were in accordance with the guidelines and regulations of the Technion's Animal welfare regulations

Note that full information on the approval of the study protocol must also be provided in the manuscript.
